# Performance of dual-energy subtraction in contrast-enhanced mammography for three different manufacturers: a phantom study

**DOI:** 10.1186/s41747-024-00516-3

**Published:** 2024-10-14

**Authors:** Gisella Gennaro, Giulia Vatteroni, Daniela Bernardi, Francesca Caumo

**Affiliations:** 1 Veneto Institute of Oncology (IOV), IRCCS, Padua, Italy; 2 Department of Biomedical Sciences, Humanitas University, Milan, Italy; 3 Radiology Department, IRCCS Humanitas Research Hospital, Milan, Italy

**Keywords:** Contrast media, Mammography, Phantoms (imaging), Radiographic image enhancement, Radiation dosage

## Abstract

**Background:**

Dual-energy subtraction (DES) imaging is critical in contrast-enhanced mammography (CEM), as the recombination of low-energy (LE) and high-energy (HE) images produces contrast enhancement while reducing anatomical noise. The study's purpose was to compare the performance of the DES algorithm among three different CEM systems using a commercial phantom.

**Methods:**

A CIRS Model 022 phantom, designed for CEM, was acquired using all available automatic exposure modes (AECs) with three CEM systems from three different manufacturers (CEM1, CEM2, and CEM3). Three studies were acquired for each system/AEC mode to measure both radiation dose and image quality metrics, including estimation of measurement error. The mean glandular dose (MGD) calculated over the three acquisitions was used as the dosimetry index, while contrast-to-noise ratio (CNR) was obtained from LE and HE images and DES images and used as an image quality metric.

**Results:**

On average, the CNR of LE images of CEM1 was 2.3 times higher than that of CEM2 and 2.7 times higher than that of CEM3. For HE images, the CNR of CEM1 was 2.7 and 3.5 times higher than that of CEM2 and CEM3, respectively. The CNR remained predominantly higher for CEM1 even when measured from DES images, followed by CEM2 and then CEM3. CEM1 delivered the lowest MGD (2.34 ± 0.03 mGy), followed by CEM3 (2.53 ± 0.02 mGy) in default AEC mode, and CEM2 (3.50 ± 0.05 mGy). The doses of CEM2 and CEM3 increased by 49.6% and 8.0% compared with CEM1, respectively.

**Conclusion:**

One system outperformed others in DES algorithms, providing higher CNR at lower doses.

**Relevance statement:**

This phantom study highlighted the variability in performance among the DES algorithms used by different CEM systems, showing that these differences can be translated in terms of variations in contrast enhancement and radiation dose.

**Key Points:**

- DES images, obtained by recombining LE and HE images, have a major role in CEM.
- Differences in radiation dose among CEM systems were between 8.0% and 49.6%.
- One DES algorithm achieved superior technical performance, providing higher CNR values at a lower radiation dose.

**Graphical Abstract:**

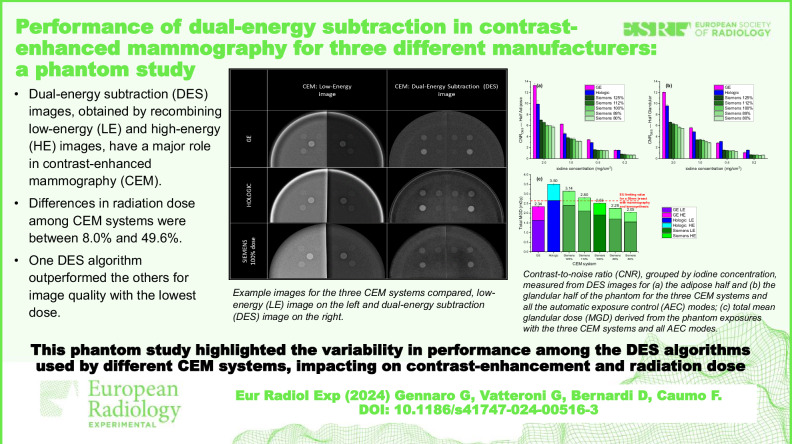

## Background

Contrast-enhanced mammography (CEM) is a dual-energy technique, which consists of acquiring pairs of mammography images, called low-energy (LE) and high-energy (HE) images, at least two minutes after intravenous administration of iodinated contrast agent to the patient. LE and HE images are recombined to generate a new image, called the following dual-energy subtraction (DES) image [1].

Compared with the soft tissues of which the breast is composed, iodine has a higher atomic number, which increases its ability to attenuate x-rays, with an absorption peak, called the *k*-edge, at the energy of 33.2 keV. The LE image is obtained by exposing the breast to an x-ray spectrum with photons mostly below the *k*-edge of iodine, and the resulting image is a standard mammogram, while the HE image is obtained with an x-ray spectrum just above the *k*-edge of iodine to maximize photoelectric absorption. The HE image is nondiagnostic but, recombined with the LE image, produces the DES image, which shows contrast uptake by any breast lesions while reducing the anatomical noise of the surrounding background [1, 2].

CEM has demonstrated efficacy in preoperative staging [3–5], monitoring of neoadjuvant therapy [6, 7], and workup of screening recalls [8–10], especially in dense breasts [11, 12]. It has also demonstrated comparable performance to breast MRI as a screening tool for women at increased risk for breast cancer, but more studies are ongoing to prospectively investigate its clinical role [13].

From a technical point of view, CEM has been developed by different manufacturers by upgrading their existing mammography equipment to incorporate dual-energy capability, each using different approaches. As a consequence, mammography units from different vendors differ fundamentally in key aspects such as x-ray source, filtering, and detector characteristics, which affect both image quality and radiation dose. CEM optimization involves careful selection of filtration and fine-tuning of technical factors for LE and HE images, *i.e*., automatic exposure control (AEC) optimization, as well as the development of recombination algorithms producing DES images while maximizing contrast and minimizing artifacts [1, 13–15]. The quality of the recombination process, which effectively removes the background tissue signal while enhancing the iodine contrast, is particularly crucial for clear visualization of lesions. Variations in these algorithms among manufacturers can lead to significant differences in the appearance of the final image. Thus, physical differences between CEM units, resulting from different choices made by manufacturers, and various recombination algorithms used contribute to the substantial variability observed in DES images between different systems [16, 17].

In this phantom study, we compared the performance of the DES algorithm among three different CEM systems using a commercial test object.

## Methods

### CEM systems

The three CEM systems compared were a GE Senographe Pristina (GE Healthcare, Chicago, IL, USA), a Hologic Selenia 3Dimensions (Hologic, Bedford, MA, USA), and a Siemens Mammomat Revelation (Siemens, Forchheim, Germany). The GE Pristina employs a dual-track x-ray tube (molybdenum, Mo, and rhodium, Rh) paired with a scintillator-based flat panel detector (FPD) with a 100-µm pixel pitch. In contrast, both Hologic and Siemens systems use a single-track x-ray tube made of tungsten (W) and a photoconductor-based FPD, with pixel pitches of 70 µm and 85 µm, respectively. Various filters are used for image acquisition. The GE system employs 30-µm Mo combined with the Mo anode and 25-µm silver (Ag) combined with the Rh anode. Hologic filters consist of 50-µm Rh or Ag, while Siemens uses 50-µm Rh for LE images. HE images are acquired using unique filters: 250-µm copper (Cu) for GE, 300-µm Cu for Hologic, and 1-mm titanium (Ti) for Siemens. The GE and Hologic systems have a single AEC mode each, called AOP/STD and AutoFilter respectively, while the Siemens system has a preset AEC, described as dose level “100%” in the DICOM “Exposure Control Mode Description” tag, with a choice of four additional modes: two to increase the dose from the default mode (labeled “112%” and “125%” dose levels) and two to decrease the dose (labeled “89%” and “80%” dose levels). The technical specifications of the three CEM systems are summarized in Table 1.

**Table 1:** Main technical specifications of the three CEM systems compared

CEM system	Senographe Pristina	Selenia 3Dimensions	Mammomat Revelation
Manufacturer	GE Healthcare	Hologic	Siemens
Detector type	CsI FPD	a-Se FPD	a-Se FPD
Pixel pitch, (µm)	100	70	85
Anode, (s)	Mo or Rh	W	W
Filters			
LE	Mo or Ag	Rh or Ag	Rh
HE	Cu	Cu	Ti
N° AEC modes	1	1	5
AEC	AOP/STD	AutoFilter	Dose level 100% (default) Dose level 125% Dose level 112% Dose level 89% Dose level 80%

AOP/STD and AutoFilter are the names given by GE and Hologic to their AEC modes, respectively. Siemens CEM differentiates its five AEC modes using part of the DICOM tag “exposure control mode description”, indicating the percentage of dose to the detector compared to the default condition (100%)

*AEC* Automatic exposure control, *a-Se* Amorphous selenium, *CEM* Contrast-enhanced mammography, *CsI* Cesium iodide, *FPD* Flat panel detector, *LE* Low energy, *HE* High energy

### Phantom

**Fig. 1:**
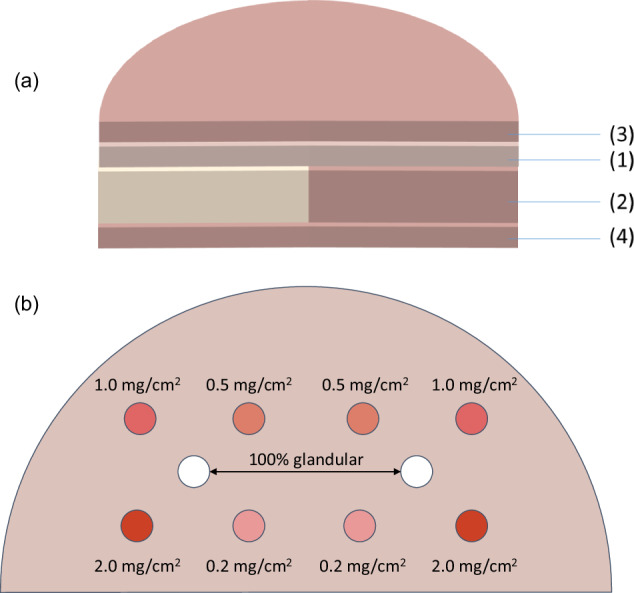
CIRS phantom Model 022 for CEM. **a** Phantom stack comprising the target plate (1) of breast-equivalent material in a 50/50 ratio of the gland to adipose tissue and a thickness of 10 mm, the contrast plate (2) of 25 mm thickness, composed half of 100% adipose material and half of 100% glandular material, the top (3) and bottom (4) plates, each 10 mm thick, composed entirely of 100% adipose material. **b** Target plate including two identical sets of five plugs, four containing iodine concentrations of 0.2 mg/cm^2^, 0.5 mg/cm^2^, 1.0 mg/cm^2^, and 2.0 mg/cm², and a central plug of 100% glandular tissue to simulate a glandular lesion. The total thickness of the phantom is 55 mm

The CIRS phantom Model 022 (Sun Nuclear, Melbourne, FL, USA) consists of four plates, as shown in Fig. 1a. The target plate (Fig. 1b) is constructed of breast-equivalent material in a 50/50 ratio of gland to adipose tissue and a thickness of 10 mm. This plate has two sets of five plugs. Four plugs within each set contain iodine concentrations of 0.2 mg/cm^2^, 0.5 mg/cm^2^, 1.0 mg/cm^2^, and 2.0 mg/cm^2^, deliberately chosen to span the clinical range of iodine concentrations. In addition, a fifth plug, placed in the center of each set, is made of 100% glandular tissue to simulate a glandular lesion. The contrast plate, 25 mm thick, consists of half 100% adipose material and half 100% glandular material. Its purpose is to validate the separation of iodine from the background over a wide range of densities. The top and bottom plates, each 10 mm thick, are composed entirely of 100% adipose material, with rounded edges to emulate the realistic shape of a compressed breast. In total, the phantom measures 55-mm thick.

In CEM mode, during the acquisition of the CIRS Model 022 phantom, the two central plugs composed of 100% glandular tissue are more visible than the plug with higher iodine concentration (2.0 mg/cm²) in the LE image. In contrast, in the DES image, the eight circular details representing the plugs at different iodine concentrations should be contrast-enhanced, while the two central plugs of 100% glandular tissue should be “invisible” [18].

### Image acquisition and analysis

For each CEM system, the CIRS Model 022 phantom was compressed with a compression force of 50 N, and three consecutive CEM studies were acquired for each available AEC mode. Four images were saved for each CEM study: the two “DICOM For Processing” LE and HE images, the “DICOM For Presentation” LE image, and the DES image.

For each study, the two DICOM For Processing LE and HE were used to obtain the technical factors (anode/filter combination, tube voltage, and exposure) selected by the AEC, and the resulting mean glandular dose (MGD) was calculated using the model proposed by Dance et al [19]. For each CEM study, the total dose was calculated as the sum of MGDs for LE and HE images.

**Fig. 2:**
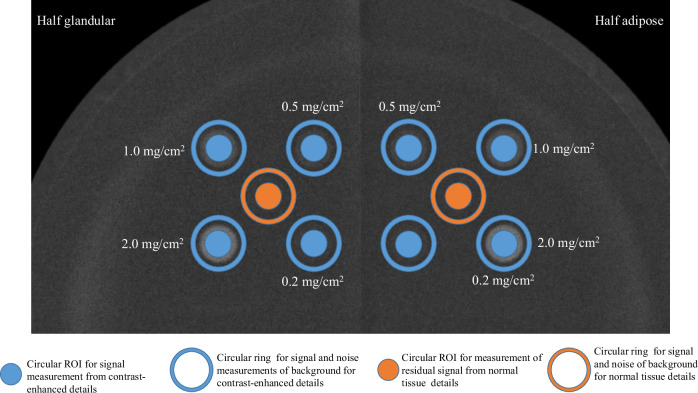
Illustration of the circular ROIs and circular rings used to measure the MPV within each target area (representing iodine concentrations and glandular plugs), as well as the MPV and SDs of the surrounding background for each target. These measurements were used to calculate the CNR. The CNR of the iodinated contrast serves as an indicator of the effectiveness of the DES algorithm in enhancing contrast, while the residual CNR in the region corresponding to the 100% glandular plugs reflects the algorithm’s ability to attenuate anatomical noise

LE and HE images were also used to measure the contrast-to-noise ratio (CNR) produced by the four plugs with different concentrations of iodine. The CNR values for this contrast-enhanced plug, along with the residual CNR in the area where the central 100% glandular plug is located, were measured from DES images for the two halves of the phantom (adipose and glandular). CNR was defined as the absolute difference in signal between an object or area of interest and the surrounding background divided by the background noise [20]. The CNR was obtained by measuring the signal strength as the mean pixel value (MPV) of a circular region of interest (ROI) placed within the target area (iodinated contrast-enhanced detail or area where the 100% glandular plug is located), and the signal and noise from the surrounding background as MPV and standard deviation (SD) of a circular ring placed around the target area. CNR was calculated by applying the following formula:

$${{{\rm{CNR}}}}=\frac{\left|{{MPV}}_{{bkg}}-{{MPV}}_{{target}}\right|}{{{SD}}_{{bkg}}}$$

where *MPV*_*target*_ is the signal produced by the target area and *MPV*_*bkg*_ and *SD*_*bkg*_ are respectively the signal and noise of the background close to the target areas. Figure 2 shows an example of a DES image with the choice of ROIs for *MPV*_*target*_, *MPV*_*bkg*_, and *SD*_*bkg*_ measurements, distinguishing between the target areas used to calculate the CNR of details with iodinated contrast and those used to assess the residual CNR of 100% glandular plugs.

### Statistical analysis

The MGD for both LE and HE images was compared among the three CEM systems and AEC modes. For each system/AEC mode, the average MGD from the three repeated acquisitions was calculated, with the maximum half-dispersion used as an estimator of the repeatability error.

Next, the CNR associated with the LE and HE images was compared. CNR values were obtained from the four plugs with different iodine concentrations for the two phantom halves. The average CNR for each system/AEC mode was calculated from three repeated acquisitions, and the maximum half-dispersion was used to estimate the repeatability error.

Finally, the performance of the DES algorithms was evaluated by considering both the total MGD (sum of the MGD of the LE and HE images) and the CNR of the DES images. For each CEM system/AEC mode, the average MGD and CNR values and the maximum half-dispersion of the three repeated acquisitions were calculated to estimate the radiation dose and an image quality metric and used for comparison.

In addition, to evaluate the performance of the removal of normal anatomical structures of the DES algorithm, the residual CNR was measured, *i.e*., the CNR measured in the two areas of phantom images corresponding to the position of the two central 100% glandular plugs.

ImageJ2 was used to measure the MPV and SD values used for CNR calculation [20].

Statistical analysis was performed by OriginPro 2020b (OriginLab Corporation, Northampton, MA, USA).

## Results

### Radiation dose

Table 2 shows the exposure parameters selected by the AEC (anode/filter combination, tube voltage, and exposure) and resulting MGD for each CEM system and all available AEC modes, separately for LE and HE images.

**Table 2:** Technical factors (anode/filter, tube voltage exposure) for the LE and HE images selected by the AEC of the three CEM systems compared

System	AEC	CEM image	A/F	Tube voltage, (kV_p_)	Exposure, (mAs)	MGD, (mGy)
GE	AOP/STD	LE	Rh/Ag	34	45.0 ± 0.9	1.64 ± 0.03
		HE	Rh/Cu	49	111.3 ± 0.4	0.70 ± 0.00
Hologic	AutoFilter	LE	W/Ag	30	156.6 ± 2.5	2.66 ± 0.05
		HE	W/Cu	49	97.6 ± 2.0	0.84 ± 0.02
Siemens	Dose level 125%	LE	W/Rh	29	215.0 ± 0.6	2.39 ± 0.02
		HE	W/Ti	49	46.8 ± 0.0	0.75 ± 0.00
	Dose level 112%	LE	W/Rh	29	191.0 ± 1.6	2.12 ± 0.02
		HE	W/Ti	49	42.3 ± 0.2	0.68 ± 0.00
	Dose level 100%	LE	W/Rh	29	172.4 ± 1.5	1.91 ± 0.02
		HE	W/Ti	49	42.3 ± 0.2	0.61 ± 0.00
	Dose level 89%	LE	W/Rh	29	154.2 ± 0.0	1.70 ± 0.01
		HE	W/Ti	49	34.4 ± 0.0	0.56 ± 0.00
	Dose level 80%	LE	W/Rh	29	138.6 ± 1.2	1.54 ± 0.01
		HE	W/Ti	49	31.7 ± 0.2	0.51 ± 0.00

The last column shows the mean value and maximum half-dispersion of the MGD associated with the two images

*AEC* Automatic exposure control, *A/F* Anode/filter, *CEM* Contrast-enhanced mammography, *HE* High energy, *LE* Low energy, *MGD* Mean glandular dose

The GE system used Rh/Ag and 34 kVp for the LE image, mirroring the settings typically used for standard mammography; for the HE image, it opted for Rh/Cu at the maximum available voltage of 49 kVp. The Hologic system, on the other hand, used W/Ag and 30 kVp for the LE image, deviating from the W/Rh and 29 kVp settings used for mammography [21], and used W/Cu with 49 kVp for the HE image. Meanwhile, the Siemens system consistently chose W/Rh and 29 kVp for the LE image and W/Ti at 49 kVp for the HE image, regardless of the AEC modes selected; variations among the five AEC modes were limited to the exposure values.

Considering the default AEC mode of the Siemens system (dose level 100%), the GE system showed the lowest MGD for LE images (1.64 ± 0.03 mGy), followed by Siemens (1.91 ± 0.02 mGy), 16.9% higher, and Hologic (2.66 ± 0.05 mGy), 62.7% higher. Notably, only the Siemens AEC mode operating at the lowest dose (dose level 80%) used a lower MGD for LE images than the GE system. For HE imaging, Siemens operated at the lowest dose (0.61 ± 0.00 mGy), followed by GE (0.70 ± 0.00 mGy), 14.1% higher, and Hologic (0.84 ± 0.02 mGy), 36.4% higher.

Considering the LE imaging of each system as comparable to standard mammography, the average dose increase attributed to HE imaging in CEM was 42.8% for GE, 31.4% for Hologic, and ranged from 31.5% to 33.1% for Siemens, depending on the AEC mode selected.

### CNR of LE and HE images

Table 3 shows the CNR (mean and maximum half-dispersion) measured on LE and HE images for the three CEM systems, and for all AEC modes. CNR values are given for each nominal iodine concentration and separately for the two halves of the phantom, adipose, and glandular.

**Table 3:** Mean CNR and maximum half-dispersion measured from the DICOM for processing (raw) LE and HE images acquired by the three CEM systems for all available AEC modes

Image type	Phantom half—iodine conc., (mg/cm^2^)	GE	Hologic	Siemens 125%	Siemens 112%	Siemens 100%	Siemens 89%	Siemens 80%
LE raw								
	Adip—2.0	4.84 ± 0.10	2.15 ± 0.04	2.56 ± 0.05	2.43 ± 0.02	2.34 ± 0.02	2.23 ± 0.01	2.16 ± 0.02
	Adip—1.0	2.63 ± 0.10	1.12 ± 0.01	1.34 ± 0.01	1.25 ± 0.03	1.25 ± 0.03	1.17 ± 0.01	1.15 ± 0.05
	Adip—0.5	1.25 ± 0.03	0.55 ± 0.01	0.64 ± 0.01	0.59 ± 0.02	0.54 ± 0.00	0.55 ± 0.02	0.54 ± 0.00
	Adip—0.2	0.56 ± 0.06	0.28 ± 0.02	0.27 ± 0.02	0.24 ± 0.06	0.26 ± 0.02	0.25 ± 0.00	0.23 ± 0.02
	Gland—2.0	3.47 ± 0.03	1.66 ± 0.00	1.82 ± 0.05	1.76 ± 0.03	1.69 ± 0.05	1.62 ± 0.02	1.56 ± 0.04
	Gland—1.0	1.91 ± 0.02	0.81 ± 0.02	1.04 ± 0.01	0.97 ± 0.03	0.95 ± 0.01	0.91 ± 0.01	0.91 ± 0.03
	Gland—0.5	1.09 ± 0.03	0.45 ± 0.01	0.52 ± 0.01	0.46 ± 0.01	0.48 ± 0.01	0.42 ± 0.01	0.40 ± 0.02
	Gland—0.2	0.55 ± 0.03	0.23 ± 0.0	0.18 ± 0.02	0.16 ± 0.01	0.18 ± 0.03	0.16 ± 0.01	0.20 ± 0.01
HE raw								
	Adip—2.0	9.51 ± 0.24	3.83 ± 0.06	3.16 ± 0.01	3.00 ± 0.04	2.77 ± 0.05	2.67 ± 0.03	2.57 ± 0.08
	Adip—1.0	4.80 ± 0.06	1.75 ± 0.05	1.41 ± 0.03	1.34 ± 0.02	1.30 ± 0.07	1.20 ± 0.05	1.20 ± 0.05
	Adip—0.5	2.72 ± 0.05	0.92 ± 0.02	0.81 ± 0.01	0.73 ± 0.02	0.71 ± 0.01	0.70 ± 0.01	0.67 ± 0.01
	Adip—0.2	1.25 ± 0.06	0.45 ± 0.03	0.39 ± 0.02	0.36 ± 0.04	0.33 ± 0.01	0.30 ± 0.04	0.28 ± 0.04
	Gland—2.0	8.72 ± 0.19	3.61 ± 0.03	2.86 ± 0.04	2.75 ± 0.03	2.56 ± 0.01	2.44 ± 0.051	2.33 ± 0.01
	Gland—1.0	4.21 ± 0.05	1.68 ± 0.01	1.40 ± 0.02	1.36 ± 0.03	1.28 ± 0.04	0.22 ± 0.02	1.18 ± 0.02
	Gland—0.5	2.63 ± 0.05	0.93 ± 0.01	0.72 ± 0.02	0.67 ± 0.01	0.65 ± 0.03	0.64 ± 0.02	0.59 ± 0.01
	Gland—0.2	1.16 ± 0.08	0.42 ± 0.03	0.34 ± 0.04	0.30 ± 0.03	0.29 ± 0.01	0.26 ± 0.03	0.28 ± 0.02

Data are reported for the two halves of the phantom, adipose (Adip) and glandular (Gland), and all iodine concentrations

*AEC* Automatic exposure control, *CEM* Contrast-enhanced mammography, *CNR* Contrast-to-noise ratio, *HE* High-energy, *LE* Low-energy

The GE system demonstrated a higher CNR than the Hologic and Siemens systems for both LE and HE images for all four iodine concentrations included in the phantom.

The ratios between the CNRs of the GE system used as a reference and those of the other systems/AEC modes are calculated in Table 4 separately for both LE and HE images, for each iodine concentration and the average value of the four concentrations.

**Table 4:** Ratio of CNR measured on DICOM for processing LE and HE images produced by the GE CEM system to that produced by the Hologic and Siemens systems (the latter for all AEC modes)

Image type	Phantom half—iodine conc., (mg/cm^2^)	GE/Hologic	GE/Siemens 125%	GE/Siemens 112%	GE/Siemens 100%	GE/Siemens 89%	GE/Siemens 80%
LE raw							
	Adip—2.0	2.2	1.9	2.0	2.1	2.2	2.2
	Adip—1.0	2.4	2.0	2.1	2.1	2.3	2.3
	Adip—0.5	2.3	2.0	2.1	2.3	2.3	2.3
	Adip—0.2	2.0	2.0	2.3	2.1	2.2	2.4
	Gland—2.0	2.1	1.9	2.0	2.1	2.1	2.2
	Gland—1.0	2.3	1.8	2.0	2.0	2.1	2.1
	Gland—0.5	2.4	2.1	2.4	2.3	2.6	2.7
	Gland—0.2	2.3	3.0	3.4	3.0	3.5	2.7
	Mean	2.3	2.1	2.3	2.2	2.4	2.4
HE raw							
	Adip—2.0	2.5	3.0	3.2	3.4	3.6	3.7
	Adip—1.0	2.7	3.4	3.6	3.7	4.0	4.0
	Adip—0.5	3.0	3.4	3.7	3.8	3.9	4.0
	Adip—0.2	2.8	3.2	3.5	3.8	4.2	4.4
	Gland—2.0	2.4	3.0	3.2	3.4	3.6	3.7
	Gland—1.0	2.5	3.0	3.1	3.3	3.4	3.6
	Gland—0.5	2.8	3.7	3.9	4.0	4.1	4.5
	Gland—0.2	2.8	3.4	3.8	4.0	4.5	4.2
	Mean	2.7	3.3	3.5	3.7	3.9	4.0

*AEC* Automatic exposure control, *CEM* Contrast-enhanced mammography, *CNR* Contrast-to-noise ratio, *HE* High-energy, *LE* Low-energy

On average, the CNR of LE images from the GE system was 2.3 times that of Hologic and 2.7 that of Siemens, while the CNR of HE images was 2.7 times that of Hologic and 3.5 that of Siemens.

### DES algorithm performance

**Fig. 3:**
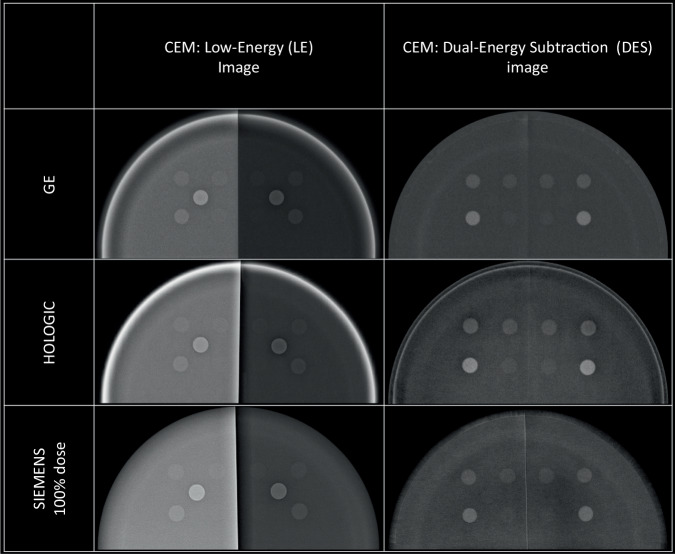
Example images for the three CEM systems compared, LE image on the left and DES image on the right. From the LE images the two halves of the phantom determined by the contrast plate (100% glandular half and 100% adipose half) can be distinguished, while they appear very similar to the DES images, indicating the overall effectiveness of the DES algorithm

A sample image for each CEM system was shown in Fig. 3, with LE images on the left and DES images on the right; for Siemens CEM the sample image was taken from the series obtained with the default AEC mode (100% dose level). It can be seen that the appearance of the phantom is different among manufacturers in both LE and DES images.

Table 5 shows the CNR (mean and maximum half-dispersion) measured on DES images for the three CEM systems, and for all AEC modes. CNR values are given for each nominal iodine concentration and separately for the two halves of the phantom, adipose, and glandular. The lower part of the table shows the ratios between CNRs obtained from the GE system used as a reference and those obtained from the other CEM systems/AEC modes.

**Table 5:** Mean CNR with maximum half-dispersion measured from the recombined DES images for the three CEM systems and all the AEC modes available

	DES images: average CNR ± maximum half-dispersion						
Phantom half—iodine concentration, (mg/cm^2^)	GE	Hologic	Siemens 125%	Siemens 112%	Siemens 100%	Siemens 89%	Siemens 80%
Adip—2.0	13.30 ± 0.32	9.90 ± 0.66	7.01 ± 0.12	6.57 ± 0.09	6.04 ± 0.16	5.96 ± 0.10	5.74 ± 0.18
Adip—1.0	6.28 ± 0.03	4.54 ± 0.17	3.80 ± 0.17	3.61 ± 0.15	3.55 ± 0.20	1.53 ± 0.07	0.69 ± 0.01
Adip—0.5	3.44 ± 0.08	2.92 ± 0.06	1.63 ± 0.07	1.54 ± 0.04	1.53 ± 0.07	1.49 ± 0.04	1.45 ± 0.05
Adip—0.2	1.54 ± 0.12	1.54 ± 0.07	0.82 ± 0.06	0.75 ± 0.14	0.69 ± 0.01	0.64 ± 0.10	0.64 ± 0.12
Gland—2.0	12.07 ± 0.18	9.58 ± 0.30	6.61 ± 0.10	6.30 ± 0.17	6.15 ± 0.04	5.70 ± 0.15	5.53 ± 0.02
Gland—1.0	5.61 ± 0.11	4.88 ± 0.14	3.61 ± 0.09	3.44 ± 0.10	3.35 ± 0.18	3.07 ± 0.10	2.90 ± 0.10
Gland—0.5	2.82 ± 0.13	3.12 ± 0.04	1.55 ± 0.02	1.49 ± 0.06	1.41 ± 0.08	1.45 ± 0.04	1.28 ± 0.04
Gland—0.2	1.10 ± 0.14	1.56 ± 0.03	0.70 ± 0.04	0.66 ± 0.08	0.67 ± 0.05	0.58 ± 0.08	0.64 ± 0.04

	DES images: the ratio between GE CNR and CNR by other CEM systems						
Adip—2.0	1.0 (ref)	1.3	1.9	2.0	2.2	2.2	2.3
Adip—1.0	1.0 (ref)	1.4	1.7	1.7	1.8	2.0	2.0
Adip—0.5	1.0 (ref)	1.2	2.1	2.2	2.2	2.3	2.4
Adip—0.2	1.0 (ref)	1.0	1.9	2.1	2.2	2.4	2.4
Gland—2.0	1.0 (ref)	1.3	1.8	1.9	2.0	2.1	2.2
Gland—1.0	1.0 (ref)	1.1	1.6	1.6	1.7	1.8	1.9
Gland—0.5	1.0 (ref)	0.9	1.8	1.9	2.0	1.9	2.2
Gland—0.2	1.0 (ref)	0.7	1.6	1.7	1.6	1.9	1.7
Mean		1.1	1.8	1.9	2.0	2.1	2.1

Data are shown for the two phantom halves, adipose (Adip) and glandular (Gland), and for the four iodine concentrations. The lower part of the table shows the ratios between CNRs obtained from the GE system used as a reference and those obtained from the other CEM systems/AEC modes

*AEC* Automatic exposure control, *CEM* Contrast-enhanced mammography, *CNR* Contrast-to-noise ratio, *DES* Dual-energy subtraction

**Fig. 4:**
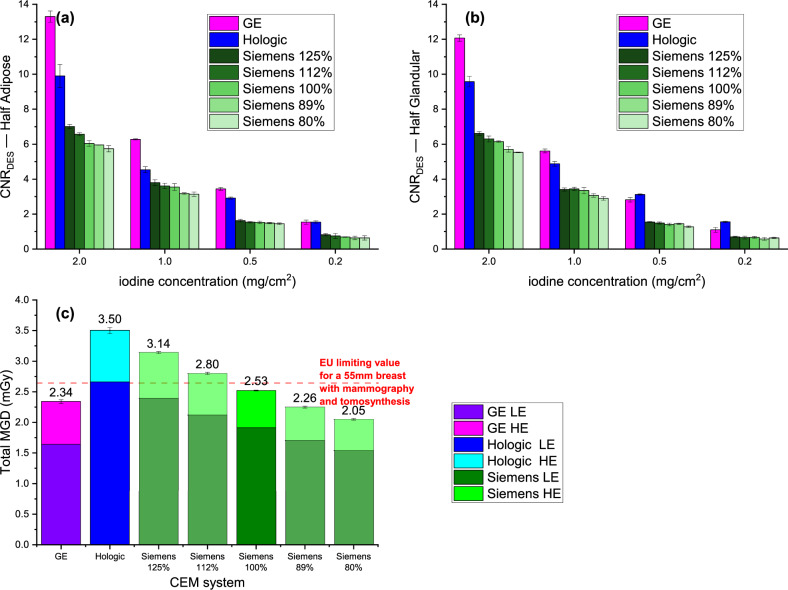
**a** CNR measured from DES images for the adipose half of phantom, grouped by iodine concentration, for all CEM systems and AEC modes. **b** CNR was measured from DES images for the glandular half of the phantom, grouped by iodine concentration, for all CEM systems and AEC modes. **c** Stacked column chart of the total MGD, calculated as the sum of MGD from both the LE and HE images, for all CEM systems and AEC modes; the red horizontal dashed line represents the EU limiting value for MGD to a 55-mm breast with mammography and tomosynthesis. The data represent the averages of three repeated acquisitions, with the error bars indicating maximum half-dispersion

Figure 4 illustrates as column plots the mean CNRs of DES images for each iodine concentration, all CEM systems, and AEC modes for the two adipose (panel a) and glandular (panel b) phantom halves, along with the total MGD (c).

Overall, the CNR values remain higher for GE images also when measured from the processed DES images, followed by Hologic and then Siemens. The largest difference was found for the two highest iodine concentrations (2.0 mg/cm^2^ and 1.0 mg/cm^2^), while the difference decreased for the lowest iodine concentrations (0.5 mg/cm^2^ and 0.2 mg/cm^2^), with very similar values for GE and Hologic.

Although the DES algorithm is designed to enhance contrast regardless of anatomical background, a lower CNR is generally expected in the glandular half than in the adipose half because of the lower signal in both LE and HE images. This trend was observed for all systems and concentrations of iodine, with the exception of the Hologic system, which showed a slightly higher CNR for the lower iodine concentrations (0.5 mg/cm^2^ and 0.2 mg/cm^2^) in the glandular half of the phantom than in the adipose half.

The Siemens system showed lower CNR values compared to the other two CEM systems across all five available AEC modes. The CNR increased as expected from the lowest dose level (80%) to the highest (125%), with relative differences ranging from a minimum of -9.8% to a maximum of 12.9% compared to the default dose level (100%).

On average, the ratio of CNR values for each iodine concentration, with the GE system taken as a reference, was 1.1 (0.7–1.4) for the Hologic system and 2.0 (1.6–2.2) for the Siemens system at the predefined dose level.

Regarding total MGD, the system that employed the lowest dose for phantom exposure was GE (2.34 ±0.03 mGy), followed by Siemens at 100% dose level (2.53 ± 0.02 mGy) and then Hologic (3.50 ± 0.05 mGy). This resulted in an increase of 8.0% for Siemens and 49.6% for Hologic over GE. However, Siemens AEC allows five different options, ranging from a maximum dose of (3.14 ± 0.01 mGy) to a minimum dose of (2.05 ±0.01 mGy), with one AEC mode using a dose level very close to GE (-3.4%) and another slightly lower than GE (-12.4%).

The GE system and the Siemens system in three of the five AEC modes keep the radiation dose below the limiting value of 2.64 mGy for a 55 mm breast established by the European Guidelines for mammography and tomosynthesis [22], while the Hologic system and the Siemens system with the two AEC modes using higher doses than the default mode exceed this value.

**Fig. 5:**
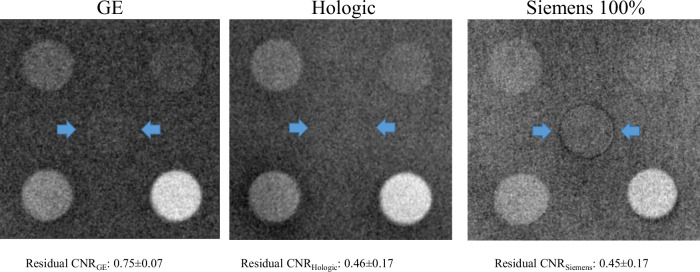
Example images for the three systems of the residual signal in the adipose half of the phantom; for each system, the contrast-to-noise value is the average value obtained from three DES images and the error is the maximum half-dispersion

Figure 5 depicts the residual signal (indicated by double arrows) for the three CEM systems (default AEC mode for Siemens) and the mean value of the residual CNR measured from the adipose phantom half, averaged over the three DES images, showing that the DES algorithms of Hologic and Siemens performed better than GE in removing the signal produced by normal breast tissue.

## Discussion

In this phantom study, several differences among manufacturers were observed in the technical parameters selected by AEC systems for image acquisition, the radiation dose employed, and the performance of DES algorithms.

Regarding radiation dose, the GE system delivered the lowest MGD (2.34 ± 0.03 mGy), while the Siemens system, operating in the default AEC mode, had a slightly higher dose (2.53 ± 0.02 mGy). In contrast, the Hologic system administered the highest dose (3.50 ± 0.05 mGy), surpassing GE by 49.6% and Siemens by 38.3%.

Analysis based on the raw images indicated that the GE system outperformed the Hologic and Siemens systems in terms of CNR for both LE and HE images in all iodine concentrations.

The GE system maintained its superior CNR performance in DES images, followed by Hologic and Siemens. However, the small differences in CNR at lower iodine concentrations suggest a decreasing advantage of the GE system as iodine content decreases. On average, the CNR ratio for each iodine concentration, using the GE system as a reference, was 1.1 for the Hologic system and 2.0 for the Siemens system at the default dose level. These ratios emphasize the superior performance of the GE system in maintaining higher CNR levels than the other systems.

Finally, residual CNR values provided insight into the effectiveness of DES algorithms in removing signals from normal breast tissue, with Hologic and Siemens outperforming GE in reducing residual signals. However, overall cancellation performance was good for all three CEM systems, with CNRs of contrast-enhanced details 1.4–3.4 times higher than the residual CNR of normal tissue.

Recently, two other research groups have published results comparing CEM systems using phantoms [16, 23]. Cockmartin et al compared the same three CEM systems and a Fujifilm Amulet Innovality system using the same phantom used in this study plus three other homemade phantoms with iodine inserts [16], while Ghetti et al used the same phantom to compare two systems included in this study (GE and Hologic), plus a Fujifilm Amulet Innovality and an IMS Giotto Class system [23]. In both papers, the focus was on the physical characterization of CEM systems and quality control. To this end, both studies used the signal difference between contrast-enhanced details and the surrounding background of DES images, plotting it as a function of iodine concentration to test its linear relationship, as initially demonstrated by Klausz et al in a poster presented at the European Congress of Radiology in 2018, which aimed to introduce the phantom CIRS Model 22 as a tool for quality control in CEM [18]. For the future development of protocols for CEM quality control, the assurance of linearity between signal difference and iodine concentration could serve as a general criterion for verifying the correct operation of DES algorithms.

In this study, CNR measured from DES images proved to be an effective metric for comparing the contrast-enhancement capabilities of different DES algorithms. Although measured from processed images, CNR remains a relevant indicator of image quality because it correlates with the detectability of contrast-enhanced details, depending on the specific detection task (*e.g*., type of lesion, contrast level, and size) [24, 25]. When used in conjunction with total MGD, CNR provides a comprehensive assessment of performance differences between DES algorithms.

However, it should not be forgotten that comparisons between systems based on phantom studies are usually more sensitive than those based on clinical data; therefore, despite the found differences in CNR between systems, the clinical relevance of these differences should be demonstrated through appropriately designed clinical performance studies.

This study has limitations. First, the phantom used represents a single breast thickness/composition, which may not capture the full range of potential differences between CEM systems in various breast thicknesses and compositions. Second, the comparison of DES algorithms was limited to three CEM systems, while other commercial CEM systems were not evaluated. Finally, since this is a phantom study, the clinical relevance of the observed CNR differences needs to be further validated in the clinical setting.

In conclusion, this phantom study demonstrated that, among the three CEM systems compared, the DES algorithm of one system performed better than the other two, providing a higher CNR at a lower dose.

## Data Availability

The datasets analyzed during the current study are available in the Zenodo repository 10.5281/zenodo.10996789.

## Abbreviations

AEC: Automatic exposure control
CEM: Contrast-enhanced mammography
CNR: Contrast-to-noise ratio
DES: Dual-energy subtraction
FPD: Flat panel detector
HE: High-energy
LE: Low-energy
MGD: Mean glandular dose
MPV: Mean pixel value
SD: Standard deviation
